# PERSONALITY TRAITS AS PREDICTORS OF BURNOUT IN SANDWICH GENERATION CAREGIVERS

**DOI:** 10.1093/geroni/igae098.2502

**Published:** 2024-12-31

**Authors:** Catherine Ju, Sabine Lohmar, Erika Fenstermacher, Montgomery Owsiany, Barry Edelstein

**Affiliations:** West Virginia University, Morgantown, West Virginia, United States; West Virginia University, Morgantown, West Virginia, United States; West Virginia University, Morgantown, West Virginia, United States; West Virginia University, Morgantown, West Virginia, United States; West Virginia University, Morgantown, West Virginia, United States

## Abstract

Informal caregiving increases the risk for burnout (i.e., emotional exhaustion, detachedness in caregiving relationships, reduced sense of accomplishment through helping). Sandwich generation caregivers, individuals who provide care for their children and older parents, face unique challenges and experience greater burnout than individuals caring for children alone. However, little research has examined what factors are associated with outcomes such as burnout in sandwich generation caregivers. Furthermore, studies on sandwich generation caregivers rarely utilize a resiliency-based approach. This study aimed to examine whether Big Five personality traits (i.e., openness, conscientiousness, extraversion, agreeableness, neuroticism) predicted burnout in sandwich generation caregivers (N = 102). A hierarchical regression model controlling for demographics, R² =.007, F(3, 98) =.222, p =.881, indicated that openness, conscientiousness, agreeableness, and neuroticism explained an additional 32.9% of the variance. This change in R² was significant, F(4, 94) = 11.628, p <.001, with openness (β = -.215), conscientiousness (β = -.265), and neuroticism (β =.257) the only significant independent predictors, R² =.336, F(7, 94) = 6.781, p <.001. In a different regression model, which controlled for depression, neuroticism was no longer a significant independent predictor. These results suggest that sandwich generation caregivers with higher levels of openness and conscientiousness and lower levels of neuroticism may experience lower levels of burnout. Findings from this study enhance our understanding of sandwich generation caregivers and what personality traits may serve as resiliency factors towards decreasing burnout. Further research can examine modifiable elements of these traits to increase resiliency.

